# Unveiling the evolutionary history of lingonberry (*Vaccinium vitis-idaea* L.) through genome sequencing and assembly of European and North American subspecies

**DOI:** 10.1093/g3journal/jkad294

**Published:** 2023-12-24

**Authors:** Kaede Hirabayashi, Samir C Debnath, Gregory L Owens

**Affiliations:** Department of Biology, University of Victoria, 3800 Finnerty Road, Victoria, BC V8W 2Y2, Canada; Agriculture and Agri-Food Canada, St.John's Research and Development Centre, 204 Brookfield Road, St. John’s, Newfoundland and Labrador L A1E 0B2, Canada; Department of Biology, University of Victoria, 3800 Finnerty Road, Victoria, BC V8W 2Y2, Canada

**Keywords:** lingonberry, partridgeberry, mountain cranberry, *Vaccinium vitis-idaea* ssp. *vitis-idaea*, *V. vitis-idaea* ssp. *minus*, genome assembly

## Abstract

Lingonberry (*Vaccinium vitis-idaea* L.) produces tiny red berries that are tart and nutty in flavor. It grows widely in the circumpolar region, including Scandinavia, northern parts of Eurasia, Alaska, and Canada. Although cultivation is currently limited, the plant has a long history of cultural use among indigenous communities. Given its potential as a food source, genomic resources for lingonberry are significantly lacking. To advance genomic knowledge, the genomes for 2 subspecies of lingonberry (*V. vitis-idaea* ssp. *minus* and ssp. *vitis-idaea* var. ‘Red Candy’) were sequenced and de novo assembled into contig-level assemblies. The assemblies were scaffolded using the bilberry genome (*Vaccinium myrtillus*) to generate a chromosome-anchored reference genome consisting of 12 chromosomes each with a total length of 548.07 Mb [contig N50 = 1.17 Mb, BUSCO (C%) = 96.5%] for ssp. *vitis-idaea* and 518.70 Mb [contig N50 = 1.40 Mb, BUSCO (C%) = 96.9%] for ssp. *minus*. RNA-seq-based gene annotation identified 27,243 and 25,718 genes on the respective assembly, and transposable element detection methods found that 45.82 and 44.58% of the genome were repeats. Phylogenetic analysis confirmed that lingonberry was most closely related to bilberry and was more closely related to blueberries than cranberries. Estimates of past effective population size suggested a continuous decline over the past 1–3 MYA, possibly due to the impacts of repeated glacial cycles during the Pleistocene leading to frequent population fragmentation. The genomic resource created in this study can be used to identify industry-relevant genes (e.g. anthocyanin production), infer phylogeny, and call sequence-level variants (e.g. SNPs) in future research.

## Introduction

*Vaccinium vitis-idaea* L., commonly known as lingonberry, partridgeberry, or mountain cranberry, is an evergreen dwarf shrub that has cultural, economic, and ecological importance (Debnath and Arigundam 2020). The bright-red colored berries have been consumed among Indigenous communities in northern North America and Scandinavia as a relish and served with meat or fish in traditional meals (Moerman 2010; Vaara *et al*. 2013). Berry picking has been a cherished cultural practice, and nowadays people commonly preserve berries as jams that are becoming more readily available commercially (e.g. Arctic Lingonberry; https://www.arcticlingonberry.fi/). A growing body of research suggests that lingonberry fruits and leaves have medicinal benefits to human health such as anticancer, cardioprotective, and neuroprotective properties (Ferlemi and Lamari 2016; Kowalska 2021). Despite a long history of utilization as a culturally important food source and its recognized health benefits, the domestication of lingonberry is at its infancy in North America.

**Fig. 1.**
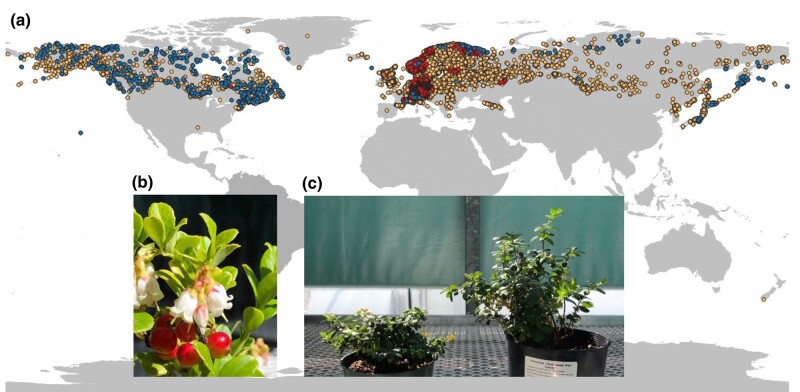
a) Worldwide distribution of *V. vitis-idaea* L. (GBIF 2023). Dots represent occurrence records registered as follows: *V. vitis-idaea* ssp. *minus* (blue), *V. vitis-idaea* ssp. *vitis-idaea* (red), and *V. vitis-idaea* L. ssp. unidentified (yellow). b) *V. vitis-idaea* ssp. *vitis-idaea* flowers and fruits. c) *V. vitis-idaea* ssp. *minus* (left) and ssp. *vitis-idaea* var. ‘Red Candy’ (right) grown in the greenhouse.

Being an evergreen boreal forest understory species, lingonberry propagates vegetatively by forming mat-like clonal communities through rhizomes (Hjalmarsson and Ortiz 1998) or sexually through seeds that are primarily insect pollinated (Jacquemart and Thompson 1996). The species has 2 recognized subspecies (ssp.) based on their geographical origin: *V. vitis-idaea* ssp. *minus* and ssp. *vitis-idaea*, and the species is widely distributed in the circumpolar region (Debnath and Arigundam 2020; Fig. 1a). The European subspecies, ssp. *vitis-idaea*, currently has active breeding programs with more than a dozen of cultivars available for commercial production, with improved yield and berry size (Penhallegon 2009). The North American ssp. *minus*, on the other hand, is considered a wild plant with little breeding efforts taken place. The 2 subspecies are distinguishable based on several morphological differences as well as genetic differences (Garkava-Gustavsson *et al*. 2005; Debnath 2007; Debnath and Arigundam 2020). The extent of genomic differences between the 2 subspecies has not been studied before, and it is somewhat unclear whether they occur sympatrically in the overlapping ranges.

Long-read sequencing technology has fueled exponential growth in the assembly of plant genomes (Marks *et al*. 2021); there are at least 1,368 unique flowering plant species genomes assembled at higher than scaffold level [NCBI search terms: “Magnoliopsida (flowering plants)” “scaffold+”, by Nov 9th, 2023], and this number is likely underestimated. The use of long reads has been particularly relevant for plant genomes due to their high repeat proportion and propensity for polyploidy. Within *Vaccinium*, high-quality genomes have been assembled for 9 species (Colle *et al*. 2019; Diaz-Garcia *et al*. 2021; Wu *et al*. 2021; Yu *et al*. 2021; Cui *et al*. 2022; Kawash *et al*. 2022; Yang *et al*. 2022; Mengist *et al*. 2023), as well as a pangenome project for cultivated blueberry and cranberry involving 32 cultivars has been completed (Yocca *et al*. 2023). In contrast, lingonberry's genomics is understudied; only a handful of genetic, chloroplast, or mitochondrial genomic research has been conducted (Garkava-Gustavsson *et al*. 2005; Debnath 2007; Gailīte *et al*. 2020; Kim *et al*. 2020; Tian *et al*. 2020). This study aimed to provide useful genomic resource to the lingonberry community, through genome assembly of the 2 distinct subspecies: *V. vitis-idaea* ssp. *vitis-idaea* and ssp. *minus*. The resources created from the study will be publicly available, in the hope of furthering our understanding of lingonberry evolution and aiding the future breeding efforts by accelerating the molecular screening of lingonberry cultivars.

## Materials and methods

### Plant material

The clones of a commercial lingonberry plant (*V. vitis-idaea* L. ssp. *vitis-idaea* var. ‘Red Candy’) were obtained from Lochside nursery (Victoria, BC) in September 2021 and July 2022 and kept in the greenhouse, designated as LC1 and LC2, respectively. Since LC1 and LC2 were clones of the same line, they should be genetically identical, but we had used separate identifiers for each. The wild lingonberry clone (*V. vitis-idaea* L. ssp. *minus*) designated as LW1, originally collected from Baie-Trinite, Quebec, Canada (latitude: 49°25′N; longitude: 67°18′W; Debnath 2007), was obtained from collaborators at Agriculture and Agri-Food Canada St. John's Research and Development Centre, NL, and kept in the greenhouse. The 3 accessions were vouchered at the University of Victoria herbarium collection: LC1 = UVIC 48749, LC2 = UVIC 48750, LW1 = UVIC 48751, respectively.

### High-molecular-weight DNA extraction

Young and mature shoots were excised from each subspecies (LC1, LW1). The leaves (1–2 g dry weight) were collected and wiped with 70% ethanol prior to extractions. The sterilized leaves were flash frozen in liquid nitrogen and ground into fine powder using mortar and pestle (∼5 min). High-molecular-weight (HMW) DNA was extracted using Nucleobond HMW DNA extraction kit (Takara Bio) following the manufacturer's protocol, with double the amount of starting material and the buffers accordingly. The DNA was then size selected using SRE-XS kit or SRE kit (Circulomics) to remove fragments smaller than 10 or 25 kb, respectively.

### RNA extraction

Total RNA was extracted for the commercial lingonberry clones, LC1 or LC2 from 5 tissue types: young expanding leaf (LC1), flower (LC2), unripe berry (greenish white; LC2), ripe berry (red; LC2), and rhizome (LC2). Note that the rhizome was technically an underground shoot, but it did not have green leaves. The root-equivalent tissue could not be sampled due to soil contaminations and difficulty in extracting enough root mass without killing the plant. For leaf and flower samples, modified CTAB protocol was used to isolate RNA (Muoki *et al*. 2012; Yoshida *et al*. 2015). For rhizome, Spectrum Plant Total RNA Kit (Sigma) was used. For berries, modified CTAB protocol optimized for bilberry was used (Jaakola *et al*. 2001). Due to low recovery of pure RNA, the unripe and ripe berries were combined to make up 1 berry sample, resulting in the total of 4 RNA samples prepared for sequencing.

### Sequencing

For long-read sequencing with Oxford Nanopore Technologies (ONT), sequencing libraries were prepared with the Ligation Sequencing Kit (SQK-LSK110 or SQK-LSK114) and they were sequenced on MinION Flow Cell R9.4.1 (FLO-MIN106D) or R10.4.1 (FLO-MIN114), respectively, following manufacturer's protocols. For LC1, 1 each of the R9.4.1 flow cell and R10.4.1 flow cell was used. For LW1, 3 R10.4.1 flow cells were used. All the raw output FAST5 reads were then basecalled by the Guppy basecalling software v6.1.2+e0556ff (https://nanoporetech.com/) and minimap2 v2.22-r1101 (Li 2018) using super accurate or “sup” model (-c dna_r9.4.1_450bps_sup.cfg). For reads generated with R10.4.1 flow cells, the reads were further duplex basecalled according to the Guppy Duplex-basecalling pipeline v6.3.8+d9e0f64 (https://nanoporetech.com/). In brief, raw FAST5 files were basecalled using the “fast” model (dna_r10.4_e8.1_fast.cfg), and the duplex candidates were listed as read–pair candidates. Those reads were then duplex basecalled by Guppy-duplex. The remaining reads were identified on the simplex reads already basecalled by “sup” model (dna_r10.4_e8.1_sup.cfg) using a custom perl script (see git repository; “filter_fastq.pl”), and finally the duplex basecalled reads were combined with the duplex-filtered simplex reads. The generated FASTQ files were concatenated as a single raw read output for the downstream procedures. Note that the raw basecalled reads were filtered by the mean >Q10 prior to concatenating. ONT sequencing and basecalling procedures are summarized in Table 1. For short-read sequencing, PCR-free whole-genome sequencing libraries were prepared and sequenced on an Illumina NovaSeq in paired-end mode, targeting 75 M individual reads per sample. The RNA library was prepared by PolyA+ mRNA Library Construction service provided and sequenced on Illumina NovaSeq paired-end mode, targeting 50 M reads per sample. Both RNA and DNA libraries were sequenced using paired-end 150 bp reads. The raw output FASTQ files were visually quality checked with fastqc v0.11.9 (Andrews 2019).

**Table 1.** ONT sequencing and basecalling methods used for commercial (LC1) and wild (LW1) lingonberry samples.

Sample	No. of flow cells used	Flow cell ver.	Flow cell code	Library kit	Simplex/duplex	Basecalling software	Basecalling mode
LC1	1	R9.4.1	FLO-MIN106D	SQK-LSK110	Simplex	Guppy (v6.1.2+e0556ff)+minimap2 v2.22-r1101	Super accurate “sup” (dna_r9.4.1_450bps_sup.cfg)
	1	R10.4.1	FLO-MIN114	SQK-LSK114	Simplex	Guppy (v6.1.2+e0556ff)+minimap2 v2.22-r1101	Super accurate “sup” (dna_r9.4.1_450bps_sup.cfg)
					Duplex (∼7%)	Guppy Duplex-basecalling pipeline v6.3.8+d9e0f64	NA
LW1	3	R10.4.1	FLO-MIN114	SQK-LSK114	Simplex	Guppy (v6.1.2+e0556ff)+minimap2 v2.22-r1101	Super accurate “sup” (dna_r9.4.1_450bps_sup.cfg)
					Duplex (∼9%)	Guppy Duplex-basecalling pipeline v6.3.8+d9e0f64	NA

### Assembly and polishing

For LC1 assembly, the filtered ONT reads were used to assemble the initial draft assembly with SmartDenovo v1.4.0 (Liu *et al*. 2021) with default parameters (smartdenovo.pl -c 1) and was polished 3 times using NextPolish v1.4.0 (Hu *et al*. 2020). The assembly was further polished with Illumina reads 3 times using Pilon v1.24 (Walker *et al*. 2014). In brief, the raw FASTQ paired-end reads were first filtered and trimmed using Trimmomatic v0.39 (Bolger *et al*. 2014; parameters used are ILLUMINACLIP:TruSeq3-PE.fa:2:30:10:2:True SLIDINGWINDOW:4:15 LEADING:3 TRAILING:3 MINLEN:36). The successfully paired reads were aligned to the long-read polished draft genome by BWA mem v0.7.17 (Li 2013), then sorted and indexed with samtools v1.10 (Danecek *et al*. 2021) prior to polishing with Pilon for a total of 3 rounds with default parameters. Lastly, haplotigs and other redundant contigs were removed using purge_haplotigs v1.1.2 (parameters -l 5 -m 42 -h 95 -j 70 -s 70; Roach *et al*. 2018). For LW1 assembly, raw ONT reads were corrected and trimmed with Canu v2.2 (Koren *et al*. 2017) and then assembled by SmartDenovo with default parameters (smartdenovo.pl -c 1). The draft assembly was similarly polished with ONT reads using NextPolish 3 times, with Illumina reads 3 times using Pilon (same parameters as LC1), and haplotigs were removed using purge_haplotigs (parameters -l 5 -m 40 -h 95 -j 70 -s 70). Note that each polishing step was done 3 rounds to ensure the error-prone reads from ONT were corrected while avoiding overpolishing (Chen *et al*. 2021). The de novo assembled genome was then scaffolded to chromosomes based on mapping contigs to the bilberry genome (Wu *et al*. 2021), using Ragtag v2.1.0 (Alonge *et al*. 2019). We did not enable the “correction” mode on Ragtag, meaning it was not looking for potential misassemblies in the de novo assembled contigs because “misassemblies” may represent genome structure differences between bilberry and lingonberry. Importantly, since both subspecies’ genomes were scaffolded from the same reference, structural variation between the 2 genomes may be missed. The final genome assembly was assessed for contiguity (N50, N90 values), per-base accuracy (Quality Value (QV) score or consensus accuracy, error rate), and completeness [Benchmarking sets of Universal Single-Copy Orthologs (BUSCO)] using BBMap v38.86 (Bushnell 2014), Merqury meryl v1.4 (Rhie *et al*. 2020), and BUSCO v5.1.2 with the following parameters: --lineage_dataset eudicots_odb10, --mode genome (Simão *et al*. 2015; Manni *et al*. 2021), respectively.

### Gene and transposable element annotation

We performed evidence-based gene annotations following the advice from the unpublished work (Freedman AH, Thomas G, Sackton TB, personal communication from https://github.com/harvardinformatics/GenomeAnnotation), which is particularly relevant for nonmodel species that lacks reliable gene models. After adapter trimming of Illumina RNA-seq reads with Trimmomatic v0.39 with parameters same as DNA (Bolger *et al*. 2014), the quality of reads was visually checked with fastqc, making sure that there was no sequence bias or decline in read quality throughout. Additionally, published transcriptome data from *V. vitis-idaea* var. ‘Sunna’ (green, white, and red berries) were added to the data set (Tian *et al*. 2020). The reads were then aligned to the scaffolded genome including all contigs using Hisat2 v2.2.1 with default parameters (Kim *et al*. 2019). Following alignment, transcript assembly was performed using StringTie v2.1.5 with default parameters (Pertea *et al*. 2015), and the transcripts were stored as structural definition file. Gene features [i.e. untranslated regions (UTRs), exons, introns, genes, and mRNAs] were then predicted on the assembled transcripts using TransDecoder v5.5.0 (Haas 2023). The longest ORF prediction (command: TransDecoder.LongOrfs) was run with -S option. A blastp reference library was prepared with *Arabidopsis* and *Vaccinium* known proteins from the UniProt database, to retain homologous hits on ORFs even if they did not exceed the coding likelihood scores used to filter ORF candidates in the preceding steps. We used *Arabidopsis* and *Vaccinium* protein databases because *Arabidopsis* is the most well-annotated flowering plant with gene models available in eudicots, and *Vaccinium* database was the closest published protein gene models to lingonberry, in the hope to discover berry-specific genes. Finally using this information, genes were predicted (command: TransDecoder.Predict) with the parameter --retain_blastp_hits. In cases where there were isoforms (genes of same genomic position, slightly different splicing pattern) or overlapping genes (splicing variants or conflicting candidate gene models), the longest gene hit was chosen as the best candidate sequence. The completeness of the predicted genes was assessed with BUSCO with the following parameters: --lineage_dataset eudicots_odb10, --mode protein (Simão *et al*. 2015; Manni *et al*. 2021).

Transposable element (TE) annotation was done following the Extensive de novo TE annotator pipeline v2.0.0 (Ou *et al*. 2019) with sensitive mode. In brief, candidate TEs were identified using LTR-Finder (Xu and Wang 2007; Ou and Jiang 2019), LTRharvest (Ellinghaus *et al*. 2020), LTR_retriever (Ou and Jiang 2018), TIR-Learner (Su *et al*. 2019), generic repeat finder (Shi and Liang 2019), and HelitronScanner (Xiong *et al*. 2014), followed by RepeatModeler (Flynn *et al*. 2020) to find any missed TEs due to structural-based methods. Finally, the combined repeat libraries were filtered so that coding sequences (CDS) from my transcript-based gene annotation did not get masked by repetitive regions (parameters: --cds, --exclude). Additional filters to effectively remove false positives were also provided at each step of combining multiple independent programs according to EDTA pipeline (Ou *et al*. 2019). To roughly map the locations of centromeres, centromere regions of the bilberry genome (*Vaccinium myrtillus*) were transferred to my lingonberry genomes using syntenic positions (Wu *et al*. 2021; Supplementary Table 1).

### Phenolic compound biosynthesis gene expression in different tissues

Phenolic compounds are important berry components for both flavor and health effects. To better understand their biosynthesis in lingonberry, enzymes in select phenolic compound and anthocyanin biosynthesis pathways were identified and then quantified using RNA-seq data in commercial lingonberry genome. Because genes that code for enzymes in anthocyanin production would be of industry and evolutionary interest, we focused our analysis on 20 enzyme-coding genes involved in the anthocyanin biosynthesis pathway, as well as closely connected pathways, in blueberry (Colle *et al*. 2019; refer to Supplementary Table 2 for the full list of enzymes analyzed). Additionally, we looked for a newly identified structural gene in anthocyanin biosynthesis pathway, glutathione transferase (GST; Eichenberger *et al*. 2023), in our assembly. We first obtained the protein sequences of structural genes of interest and aligned them against the blueberry genome annotation using BLAST (Altschul *et al*. 1990) to find which blueberry genes correspond to which enzymes. We then identified gene orthology between lingonberry and other *Vaccinium* species using OrthoFinder (Emms and Kelly 2019). Note that the tetraploid “Draper” protein sequences were kept as a full set preserving all 4 haplotypes to find a potential match in lingonberry. OrthoFinder places genes into orthogroups representing orthology. Any annotated lingonberry gene found in the same orthogroup as a blueberry gene was a potential enzyme. We then filtered this set to require that the lingonberry gene was ≥95% identical in sequence to its closest blueberry ortholog, and that it was ≥80% of length of the blueberry ortholog. In this way, we enriched for orthologs that were likely to have the same function.

Using the LC1 assembly and gene annotation file produced above as a reference, expression levels of the annotated transcripts/genes were estimated by Hisat2 with -A, -G and -e option (Kim *et al*. 2019). The abundance estimate from the 7 transcript data sets (i.e. LC1 leaf, LC2 rhizome/flower/berry, and green/white/red berry from Tian *et al*. 2020) was reported in the units of FPKM for each data set, corresponding to fragments per kilobase of transcript per million mapped fragments (Zhao *et al*. 2021).

### Genomic divergence between subspecies

To calculate pairwise nucleotide divergence between the 2 lingonberry subspecies genomes, the 12 scaffolded chromosomes were aligned using minimap2 v 2.24-r1122 (Li 2018, 2021) with LW1 scaffolded genome as a reference and LC1 scaffolded genome as a query (default parameters: -ax ams5 --cs=long). Following data format conversions (paftools.js sam2paf | view -f maf), the alignment file was filtered to remove duplicate alignments and the pairwise divergence was calculated per 10 kb windows using maffilter v1.3.1 (Dutheil *et al*. 2014) parameters: Subset(remove_duplicates=yes, keep=no), MinBlockLength(min_length=1000), WindowSplit (preferred_size=10000, align=ragged_left), SequenceStatistics (Pairwise Divergence)). The program computes the number of base pair mismatches based on the alignment file and reports this value as the divergence in % mismatch in the specified window size. Additionally, to explore the presence of structural variations and basic sequence variations, Synteny and Rearrangement Identifier v1.5 (Goel *et al*. 2019) was used on the aligned chromosomes with default parameters. We note that since both genomes were scaffolded using the same bilberry reference genome, overall synteny was likely inflated and we might not be capturing all structural variation between the species.

### Demographic history estimate

In order to investigate the past population history of lingonberry subspecies, we utilized multiple sequentially Markovian coalescent model (MSMC2; Schiffels and Wang 2020) and pairwise sequentially Markovian coalescent model (PSMC; Li and Durbin 2011). MSMC2 requires that the analyzed populations are mapped to the same reference genome. For the purpose of comparing the 2 methods in parallel, we chose to use LW1 as a reference genome for both subspecies because of better contiguity and base pair accuracy than LC1. To first calculate the effective population size (*N*_e_) of each subspecies, the paired Illumina reads were mapped to the LW1 genome using BWA mem v0.7.17 (Li 2013) with default parameters. PCR and optical duplicates were then removed using GATK Picard v2.23.2 “MarkDuplicates” function (Van der Auwera and O’Connor 2020). The mappable heterozygous variant sites were identified separately for each chromosome per subspecies following bamCaller.py in MSMC2 v2.1.3 (Schiffels and Wang 2020). In brief, SNPs were first called using bcftools v1.16 (Danecek *et al*. 2021) with the command “mpileup” and “call” with the parameters -q 20 -Q 20 -C 50 and -c -V indels, respectively. The results were then filtered and organized based on read coverage (mean coverage set to 38 for LW1, 37 for LC1; filtering applied is the minimum of ×1/2 mean coverage to the maximum of ×2 mean coverage). An additional mappability mask was generated to avoid calling variants from significantly repetitive regions using GenMap v1.3.0 (Pockrandt *et al*. 2020) with the parameter -K 30 -E 2. For PSMC inputs, SNPs were similarly called using bcftools “mpileup” and “call” with the same parameters as above, and the results were filtered with the minimum of ×1/3 and maximum of ×2 mean coverage, as recommended (Li and Durbin 2011). No repeat mappability mask was considered in PSMC analysis. When running the models, a generation time of 5–10 years was chosen based on a prior experiment observing minimum of 8 years required to consider a seedling fully reproductive (Hjalmarsson and Ortiz 1998) and considering the woody shrub's natural age of first flowering (Ritchie 1955). However, given the potential for reproduction after first maturity, we recognize that this might underestimate the average reproductive age of the natural population. A mutation rate of 3 × 10^9^ substitutions per generation from *Arabidopsis thaliana* was used (Exposito-Alonso *et al*. 2018).

### Phylogenetic tree construction

Phylogenetic trees were constructed using 2 different approaches. The first approach followed the default pipeline provided using OrthoFinder v2.5.4 (Emms and Kelly 2019). In brief, a total of 11 species protein sequences in amino acid fasta format were collected from published studies: 8 *Vaccinium* species: 2 *V. vitis-idaea* subspecies from this study, *Vaccinium corymbosum* var. ‘Draper’ v1.0 first 12 chromosomes (Colle *et al*. 2019), *Vaccinium macrocarpon* var. ‘Stevens’ v1.0, *Vaccinium microcarpum* v1 (Diaz-Garcia *et al*. 2021), *Vaccinium oxycoccos* NJ96-20 v1 (Kawash *et al*. 2022), *V. myrtillus* NK2018_v1 (Wu *et al*. 2021), *Vaccinium darrowii* v1.2 (Cui *et al*. 2022), and *Vaccinium caesariense* W85-20 P0 v2 (Mengist *et al*. 2023). Kiwi fruit or *Actinidia chinensis* v3.0 (Tang *et al*. 2019) and azalea or *Rhododendron williamsianum* (Soza *et al*. 2019) were used as outgroups. The species tree was constructed based on the individual gene trees inferred from the orthologous gene groups as per OrthoFinder pipeline (Emms and Kelly 2017, 2018). For further validation using conserved genes only, single-copy BUSCO genes were extracted and aligned to infer species tree. To do that, BUSCO analysis was first performed on the collected genome assembly itself in nucleotide fasta format with --lineage_dataset eudicots_odb10, --mode genome (Simão *et al*. 2015; Manni *et al*. 2021). Then the identified single-copy genes were aligned by MAFFT v7.310, and the individual gene trees were inferred with IQ-TREE v1.5.5 (Nguyen *et al*. 2015). Outlier long branches were trimmed by TreeShrink v1.3.9 (Mai and Mirarab 2018) with default parameters. Finally, the species tree was constructed using the trimmed gene tress in Astral III v5.7.8 (Zhang *et al*. 2018). For visualization and data interpretation, both species trees were exported in Newick format and then viewed in FigTree. Trees were rooted manually to *A. chinensis*.

Additionally, divergence times were estimated following (Diaz-Garcia *et al*. 2021). In brief, single-copy BUSCO gene alignments were used as an input alignment file with RelTime as implemented in MEGA X (Tamura *et al*. 2012, 2018). *Actinidia chinensis* was set as an outgroup, and the following calibration time was used based on the average of 16 studies in TimeTree (Kumar *et al*. 2017): *Rhododendron* and *Vaccinium* (45.5–76.9 MYA). Uniform distribution was selected as the calibration density. Due to MEGA X requiring a single sequence alignment file with equal sequence length, only 743 BUSCO genes that were present in all 11 species were selected for analysis. The individually aligned BUSCO genes were concatenated to prepare the input file with seqkit concat function (Shen *et al*. 2016). Note that 7 ambiguous amino acid “J”s corresponding to isoleucine or leucine in the alignment file were manually replaced with “I”s in order to meet the requirements by MEGA X.

## Results and discussion

### Sequencing and assembly

Collectively, 35.3 Gb (∼50.0X) of clean (≥Q10) long-read data was generated (read N50 = 20.56 kb), and additional 12.42 Gb (∼37X) of short-read data was generated for the commercial subspecies, LC1. The de novo assembly resulted in 757 contigs of total length 548.004 Mb with BUSCO (Complete) = 96.6%, contig N50 = 1.170 Mb, and per-base accuracy = 99.959%. Similarly, 28.6 Gb (∼46.9X) of clean long-read data (read N50 = 23.16 kb) and 10.9 Gb (∼35X) of short-read data were generated for the wild subspecies, LW1. The final de novo assembly had 518.642 Mb of total assembly length with contig N50 = 1.400 Mb, BUSCO (Complete) = 96.8%, and per-base accuracy = 99.975% (Table 2; Supplementary Tables 3 and 4). The assembled genome sequence lengths were consistent or slightly smaller than flow cytometry estimates that measured a ∼550 Mb genome size (Redpath *et al*. 2022). Compared to the short-read only assemblies, which generally do not reach N50 of 1 Mb, our ONT-based assemblies were significantly more contiguous (Rhie *et al*. 2021), and our assembly statistics were comparable to many draft genome assemblies of similar size (Marrano *et al*. 2020; Wu *et al*. 2021; Hamilton *et al*. 2023; Zhang *et al*. 2023).

**Table 2.** Genome assembly statistics.

	De novo assembly	Haploid only	Scaffold assembly
*V. vitis-idaea* ssp. *vitis-idaea* (LC1)			
Total length (Mb)	614.857	548.004	548.071
Contig N50 (Mb)	1.028	1.170	1.170
Scaffold N50 (Mb)			43.867
No. of fragments/contigs	1358	757	757
No. of scaffolds			92
BUSCO (C%)	96.8	96.6	96.5
BUSCO (S%)	84.3	87.5	88.4
BUSCO (D%)	12.5	9.1	8.1
QV score	33.8254		
Accuracy (1-error rate)	99.959%		
Genome anchored to chr (%)			98.0
No. of genes annotated			27,243
Coding gene content (%)			7.59
TE content (%)			45.82
*V. vitis-idaea* ssp. *minus* (LW1)			
Total length (Mb)	545.497	518.642	518.704
Contig N50 (Mb)	1.309	1.400	1.400
Scaffold N50 (Mb)			42.799
No. of fragments/contigs	1030	696	696
No. of scaffolds			76
BUSCO (C%)	96.9	96.8	96.9
BUSCO (S%)	89	89.7	90.5
BUSCO (D%)	7.9	7.1	6.4
QV score	35.9577		
Accuracy (1-error rate)	99.975%		
Genome anchored to chr (%)			98.5
No. of genes annotated			25,718
Gene content (%)			7.37
TE content (%)			44.58

Note that the haploid only assembly (for a diploid genome) meant heterozygous alleles were represented as a mixed haplotype from either of the homologous copy, but not both. The allelic sequences with less confidence were purged during assembly correction based on sequence coverage (Roach *et al*. 2018).

**Fig. 2.**
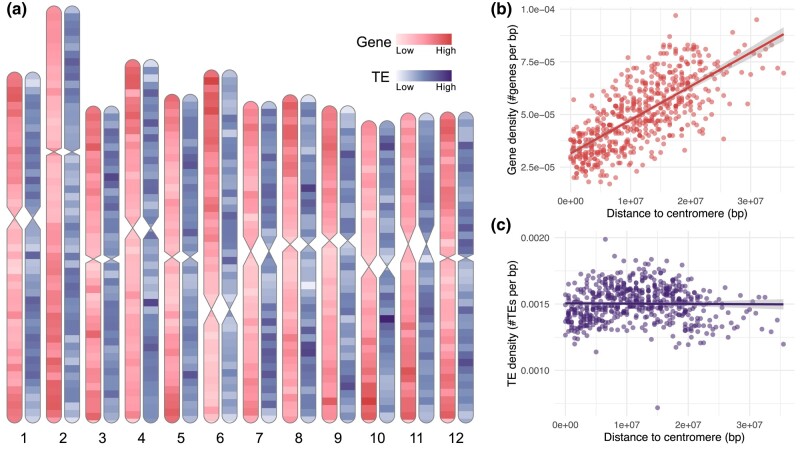
a) Gene and TE distributions in lingonberry genome (*V. vitis-idaea* var. ‘Red Candy’). b) Gene and c) TE densities by distance from centromeres. Centromere positions were approximately mapped from bilberry genome as a range, and distance was calculated to its middle value (Wu *et al*. 2021). Red shades indicate the gene density, and purple shades indicate the TE density. Genes were filtered to represent only the longest gene in case of isoforms and splicing variants present. All densities are presented as the number of feature counts per 1 Mb, except the terminal windows <1 Mb.

Scaffolding was performed by mapping to the nearest relative with a chromosome-scale genome, bilberry (*V. myrtillus*; Schlautman *et al*. 2017; Kim *et al*. 2020; Fahrenkrog *et al*. 2022), resulting in the total of 92 and 76 scaffolds, scaffold N50 = 43.867 and 42.799 Mb, and 98.0 and 98.5% of the contigs anchored to chromosomes for LC1 and LW1, respectively (Table 2). We characterized genomic differences between the subspecies using SyRI and found no major translocations, perhaps due to common scaffolding, and low levels of genome-wide divergence in sequence (Supplementary Tables 5 and 6 and Figs. 1 and 2). We recognize that reference-based scaffolding of the genome does not necessarily produce the real genome structure of lingonberry. This is because the true structural variations can be rearranged during scaffolding as the algorithm orients and places contigs based on alignment to the reference genome (Alonge *et al*. 2019). That being said, a recent study in *Eucalyptus* scaffolded ONT genomes on congeneric reference genome to study genome structure evolution and found that a very small proportion of synteny breakpoints were at contig joins, as might be expected if scaffolding was inducing false rearrangements (Ferguson *et al*. 2023). Therefore, the 2 lingonberry genomes created in this study can reasonably serve as a reference genome to identify genes, polymorphic genetic markers, and compare with related species. Future efforts could generate an unbiased scaffolding using Hi-C or optical mapping and additionally test for the amount of bias introduced by scaffolding to a related reference genome.

### Annotation

RNA-seq data were produced from leaf sample (∼7.8 Gb), rhizome (∼6.9 Gb), flower (∼11.4 Gb), and berry (∼11.7 Gb) samples in the commercial subspecies, LC1 and LC2. The 2 clones were treated as genetically identical. In addition, transcript data from a published work were added to our analysis (Tian *et al*. 2020). With the alignment of RNA reads to the assemblies, the total of 27,243 and 25,718 genes were annotated [BUSCO (C): 91.4 and 91.7%]. Excluding non-CDS (introns, UTRs, etc.), the CDS content was 7.59 and 7.37% across the genome, with the average length of 238 and 231 bp for LC1 and LW1, respectively. TEs were also annotated using multiple independent programs and found to cover 45.82 and 44.58% of the genome overall (Table 2). We observed that TE density was fairly even across the genome whereas genes were tended to locate less around putative centromeres and more on distal chromosome positions (Fig. 2). When plotting the TE distributions by different types (Supplementary Fig. 3), some differences in density across the chromosome were observed.

### The phenolic compound biosynthesis pathway in lingonberry

**Fig. 3.**
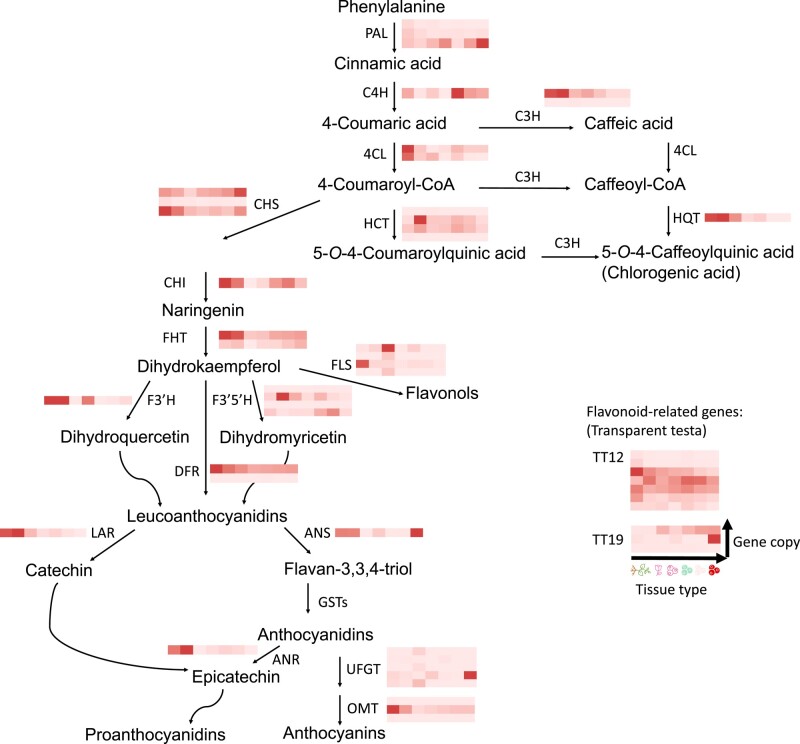
Heatmap of gene abundance related to phenolic compound biosynthesis. Columns represent sample type, and rows represent gene copies on lingonberry genome. Sample types are (from left to right) *V. vitis-idaea* var. ‘Red Candy’ rhizome, leaf, flower, berry, and var. ‘Sunna’ berries at different ripening stages; green berry, white berry, and red berry (Tian *et al*. 2020). Abundance was measured by FPKM. Note that the red color gradient was normalized within each heatmap, so comparison cannot be made across heatmaps. The enzyme pathway is based on Colle *et al*. (2019).

In this study, 49 putative phenolic compound biosynthesis-related genes composed of 20 distinct enzymes/structural gene categories were identified in lingonberry through orthology to the tetraploid commercial blueberry genome (Colle *et al*. 2019).We did not detect an ortholog of the GST gene in our annotated gene set using OrthoFinder, which uses amino acid similarity for orthology detection. When using BLAST to search our genome nucleotide sequence for the GST gene, we found a single gene (STRG.3821) with ∼96% similarity to the *V. corymbosum* GST gene, although this gene was more than 7,000 bp long, much longer than the functionally active 700 bp long GST gene (Eichenberger *et al*. 2023). This suggests significant changes to the lingonberry GST gene or errors in gene annotation. We saw a significantly increased expression of ANS and TT19 in the red berries (Fig. 3), which was consistent with their described roles in anthocyanin production and accumulation (Kitamura *et al*. 2004; Lin *et al*. 2018). Although the phenolic-related genes were expected to be highly expressed in berries compared to other tissue types, rhizome and leaf expressed C4H, HCT, HQT, CHI, FHT, F3'H, LAR, and ANS at much higher levels than the berry samples (Fig. 3). Considering various known physiological roles of phenolic secondary metabolites in plants (Albert *et al*. 2022), abundant expression of genes in vegetative tissues implied that phenolics played roles in stress tolerance.

While anthocyanins and the related phenolic compounds are the major targets of breeding due to their health benefits (Edger *et al*. 2022) and there has been efforts to build QTL maps associating genomic regions to increased anthocyanin production in commercial blueberry and cranberry (Diaz-Garcia *et al*. 2018; Montanari *et al*. 2022), the genetic basis for anthocyanin biosynthesis in lingonberry is relatively understudied. The QTL study that specifically targeted the increased anthocyanin production in blueberry suggested candidate genes including BAHD acyltransferase and UFGT to be highly correlated with the increased anthocyanin profile (Montanari *et al*. 2022). We were able to annotate 5 copies of UFGT in lingonberry genome, 1 of which was highly expressed in red berries (STRG.15162 on chromosome 4; Supplementary Fig. 4). The genomic resource created in this study could be used to find such orthologs and provide a starting point to develop a set of lingonberry-specific markers that could be useful to accelerate the breeding efforts by encouraging marker-assisted selection.

### Historical population size and origin of lingonberry

**Fig. 4.**
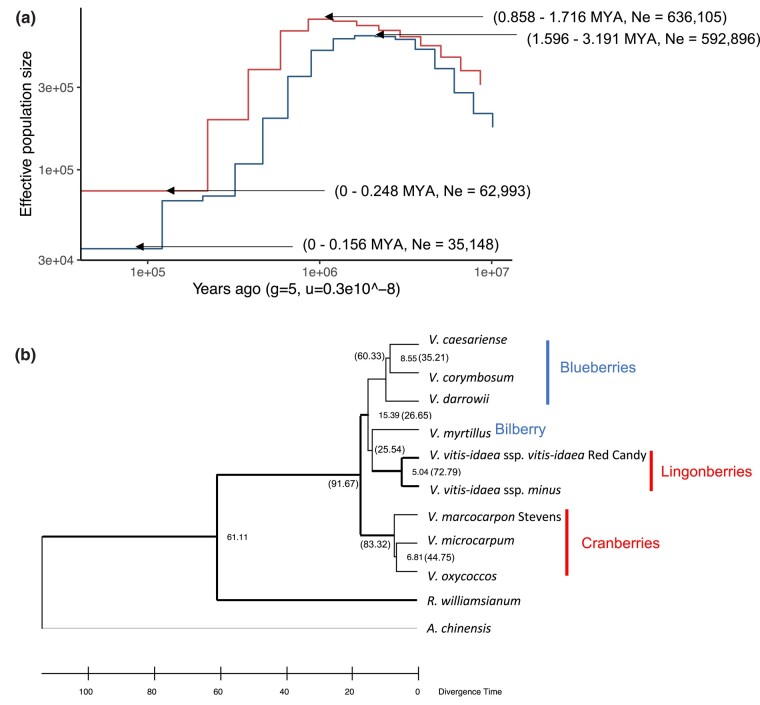
a) Past effective population size (*N*_e_) of lingonberry with MSMC2. The *N*_e_ of *V. vitis-idaea* ssp. *minus* (blue; LW1) and *V. vitis-idaea* ssp. *vitis-idaea* var. ‘Red Candy’ (red; LC1) was plotted against years before present. Both *x*- and *y*-axes were log scaled. Plots were generated with the generation time of 5 years and mutation rate of 3 × 10^9^ mutations/generation. Note the timings are presented as the range estimate from generation time of 5–10 years. b) Phylogeny of *Vaccinium* based on 2,226 conserved BUSCO genes. Thick lines indicate nodes supported by >60 STAG support values in OrthoFinder (Emms and Kelly 2018, 2019). The numbers on the selected node represent divergence time in million years (MY), calibrated at the divergence time with *Rhododendron* (45.5–76.9 MY), and the number in bracket shows the gene concordance factor (0–100) obtained from 2,226 BUSCO genes.

The genetic structures of the contemporary populations can often be shaped by the isolation history, which is especially relevant among subarctic/alpine plants that underwent past population fragmentation due to ice sheets during the Pleistocene (Hewitt 2000; Eidesen *et al*. 2013). Previous genetic studies in lingonberry revealed the impact of repeated glaciation on its contemporary patterns of genetic diversity (Debnath 2007; Eidesen *et al*. 2013). Leveraging the genome-wide variant calling along chromosomes, we were able to estimate the historical effective population size (*N*_e_) using PSMC and MSMC2. Despite current range expansions, our result indicated an ongoing population bottleneck for both European (LC1) and North American (LW1) populations. Using a generation time of 5–10 years, we estimated that LC1 and LW1 began declining in *N*_e_ around 0.8–1.7 MYA and 1.5–3.2 MYA (Fig. 4a). Lingonberry has likely undergone repetitive range contractions followed by expansion due to ice sheets advancing and receding, which may explain the population size declines over the last 1–2 MYA.

At species level, we generated a phylogeny using all the available *Vaccinium* whole-genome data. The protein sequence alignment across 8 *Vaccinium* species and 2 outgroup species resulted in the total number of 377,681 genes analyzed, of which 349,420 were categorized into 31,264 orthogroups by OrthoFinder (Emms and Kelly 2019). The mean orthogroup size was 11.2 genes, and 5,941 orthogroups were shared by all the species, of which 241 were single-copy orthogroups. Additionally, we built species trees based on 2,226 conserved single-copy BUSCO genes to confirm the congruence with the OrthoFinder result using Astral (Zhang *et al*. 2018). We found that generally there were monophyletic groups for cranberries (*V. microcarpum*, *V. oxycoccos*, and *V. macrocarpon*) and blueberries (*V. darrowii*, *V. caesariense*, and *V. corymbosum*), while bilberry (*V. myrtillus*) was identified as the closest relative of lingonberry (*V. vitis-idaea*; Fig. 4b), in agreement with the previous studies (Schlautman *et al*. 2017; Kim *et al*. 2020; Fahrenkrog *et al*. 2022). Interestingly, this suggested that there were multiple color changes of berries in *Vaccinium* lineage. Adding more species to the current tree, particularly those closely related to lingonberry and bilberry, could address whether the red berry phenotype had convergently evolved in cranberry and lingonberry lineage. Gene concordance values were generally low especially among species in the blueberry, bilberry, and lingonberry (ranging from 25 to 35). Although further analysis is required to fully understand the relationship, it implied that *Vaccinium* had high levels of incomplete lineage sorting or possibly introgression between species (Coyne and Orr 2004; Beeler *et al*. 2020).

Our time-calibrated phylogeny suggested that the 2 lingonberry subspecies diverged 5 MYA, which was similar in scale to sister species divergence times in cranberry (6.8 MYA) and blueberry (8.5 MYA). We express some caution in our exact timing because this was based on a single fossil calibration and there was a lack of fossil or geological data in the younger interspecies nodes (Kumar *et al*. 2017). Compared to previous estimates, our divergence times were consistent (Cui *et al*. 2022) or overestimated (Diaz-Garcia *et al*. 2021). Nevertheless, the relative divergence between lingonberry subspecies and other *Vaccinium* species pairs suggested that the subspecies were near the divergence level expected between species and raised questions about their taxonomic classification. Further work is needed to evaluate where crossability barriers exist between ssp. *minus* and ssp. *vitis-idaea*, although high crossability is common between recognized *Vaccinium* species (Edger *et al*. 2022). The relatively old divergence time means that the parallel population bottlenecks in both subspecies are not shared but instead are independent events.

### Conclusion

This study characterized the genomes of both lingonberry subspecies. Using these genomic resources, we identified genes likely functioning in phenolic compound biosynthesis and clarified the phylogenetic position of lingonberry. The data generated in this study will facilitate future work, such as generation of genetic markers for breeding and analysis of population structure across the species range. Further, the results encouraged scientists in the field to address novel hypotheses regarding not only the evolution of lingonberry but also the evolution of diverse edible berries in the genus *Vaccinium*.

## Supplementary Material

jkad294_Supplementary_Data

## Data Availability

This whole-genome sequencing project has been deposited at DDBJ/ENA/GenBank under the accessions JAUYVE000000000 (LC1) and JAUYVF000000000 (LW1). The raw sequences are archived in SRR25468432-50 (ONT) and SRR25477285-90 (Illumina). Full annotations and reference-mapped genome assemblies used in this manuscript can be downloaded from figshare: https://figshare.com/projects/Unveiling_the_evolutionary_history_of_lingonberry_Vaccinium_vitis-idaea_L_through_genome_sequencing_and_assembly_of_European_and_North_American_subspecies/175089. All codes used for assembly pipeline and downstream analysis are available at https://github.com/kaede0e/lingonberry_genomics. Supplemental material available at G3 online.
