# Supplementary material for: Unveiling the evolutionary history of lingonberry (Vaccinium vitis-idaea L.) through genome sequencing and assembly of European and North American subspecies
Source: G3 (Bethesda). 2023 Dec 24;14(3):jkad294. doi: 10.1093/g3journal/jkad294 (PMC10917501; doi:10.1093/g3journal/jkad294)
Supplement: jkad294_Supplementary_Data [file jkad294_supplementary_data.zip › Supplementary_Material_Legends_G3-2023-404686.docx]

**Supplemental material:**

Supplementary Table 1: Chromosome lengths and putative centromere positions on lingonberry genome. Centromere positions are putatively assigned based on bilberry genome (Wu *et al.* 2021).

Supplementary Table 2: Orthologues for proposed phenolic compound biosynthesis genes (Colle *et al.* 2019) in lingonberry annotation.

Supplementary Table 3: Oxford Nanopore MinION outputs generated from this study.

Supplementary Table 4: Illumina outputs from this study.

Supplementary Table 5: Detected structural variations between lingonberry subspecies. Reference genome was set as *Vaccinium vitis-idaea* ssp. *minus* and the query genome was *V. vitis-idaea* ssp. *vitis-idaea* var. ‘Red Candy’. Variations were detected by SyRI (Goel *et al.* 2019) on the 12 chromosomes only and unplaced contigs were not considered into analysis.

Supplementary Table 6: Detected sequence level variations between lingonberry subspecies. Reference genome was set as *Vaccinium vitis-idaea* ssp. *minus* and the query genome was *V. vitis-idaea* ssp. *vitis-idaea* var. ‘Red Candy’. Variations were detected by SyRI (Goel *et al.* 2019) on the 12 chromosomes only and unplaced contigs were not considered into analysis.

Supplementary Figure 1: Alignment between two lingonberry subspecies detected by SyRI (Goel *et al.* 2019).

Supplementary Figure 2: Pairwise divergence between *Vaccinium vitis-idaea* ssp. *minus* (LW1) and ssp. *vitis-idaea* (LC1).

Supplementary Figure 3: Scatter plots of long-terminal-repeat (LTR) and hAT terminal inverted repeat (hAT TIR) densities in comparison to gene density against the distance from the centre of chromosomes.

Supplementary Figure 4: Heatmap of phenolic compound biosynthesis related gene abundance in lingonberry. Gene abundance was measured in the unit of FPKM, and the values are z-scaled per enzyme, and then combined for visualization. Rows represent different copies of each orthologous gene in lingonberry (enzyme name_STRG-id), and columns are sample types.
